# A Pilot Study for Control of Hyperendemic Cystic Hydatid Disease in China

**DOI:** 10.1371/journal.pntd.0000534

**Published:** 2009-10-27

**Authors:** Wenbao Zhang, Zhuangzhi Zhang, Turhong Yimit, Baoxin Shi, Hasyeti Aili, Gulnor Tulson, Hong You, Jun Li, Darren J. Gray, Donald P. McManus, Jincheng Wang

**Affiliations:** 1 Xinjiang Veterinary Research Institute, Xinjiang Academy of Animal Science, Urumqi, Xinjiang, China; 2 Molecular Parasitology Laboratory, Australian Centre for International and Tropical Health and Nutrition, Queensland Institute of Medical Research Brisbane, Queensland, Australia; University of Oklahoma Health Sciences Center, United States of America

## Abstract

**Background:**

Cystic hydatid disease (CHD) is a global parasitic zoonosis caused by the dog tapeworm, *Echinococcus granulosus*. The disease is hyperendemic in western China because of poor economic development; limited community knowledge of CHD; widespread, small-scale household animal production; home killing of livestock; and the feeding of dogs with uncooked offal.

**Methodology/Principal Findings:**

A control program focusing on monthly praziquantel (PZQ) treatment of all registered dogs and culling unwanted and stray dogs has been designed to control CHD in hyperendemic areas in China. A pilot field control project in two counties (Hutubi and Wensu) in Xinjiang, China showed that after 4 years of treatment, the prevalence of dogs with *E. granulosus* was reduced from 14.7% and 18.6%, respectively, to 0%, and this caused a 90%–100% decrease of CHD in sheep born after commencement of the control program.

**Conclusions/Significance:**

The strategy aimed at preventing eggs being released from dogs into the environment by treating animals before adult tapeworms are patent can decrease *E. granulosus* transmission and considerably reduce hyperendemic CHD. Monthly treatment of dogs with PZQ and culling unwanted and stray dogs have been shown to be an efficient, highly cost-effective and practicable measure for implementation in rural communities. As a result, the Chinese Ministry of Health has launched an extensive CHD control program in 117 counties in western China using this control strategy.

## Author Summary

Cystic hydatid disease (CHD), caused by the dog tapeworm, *Echinococcus granulosus*, is hyperendemic in western China. However, until recently the disease had been grossly neglected there due primarily to a weak economy and the primitive control measures used. The situation is now changing because of China's growing economy and the availability of the cheap and effective drug praziquantel (PZQ), which makes control programs practicable through mass preventive treatment of dogs in these endemic areas. In this study, we used a strategy to prevent parasite eggs from being released into the environment by dosing dogs monthly with baited PZQ and culling unwanted and stray dogs, as a result of which infection in humans and domestic animals can be reduced considerably. We undertook a pilot control study in two counties involving a population of 255,504 in 52,300 households and 30,380 dogs in Xinjiang, China, and showed that monthly PZQ treatment of dogs is an efficient, practicable, and affordable control method for communities to reduce considerably hyperendemic CHD. As a result, the Chinese Ministry of Health has extended the control measure to 117 counties in western China.

## Introduction

Cystic hydatid disease (CHD), caused by *Echinococcus granulosus*, is hyperendemic in China, especially in western areas. In 1989, Tibet had the highest prevalence of human CHD (cases from hospital records) with 670 cases/100,000 inhabitants, followed by Qinghai (220/100,000), Ningxia (130/100,000), Xinjiang (91/100,000) and Gansu (22/100,000) [1]. More recent surveys using ultrasound and X-ray analysis showed that 3%–12% of Tibetans had hydatidosis (both CHD and alveolar hydatid disease cases) in Ganzi, Sichuan Province [2], and 1%–10% in south Qinghai Province [3],[4], Ningxia [5], and Gansu [6]. In these regions as well, more than 50% of 170 million domestic animals (58% being sheep) were infected with CHD [1]. However, animal production is characterized by its small scale; every household has about ten sheep per household in agricultural areas and 100–300 sheep per household in remote animal farming areas [1],[7]. Home killing of animals for family meat consumption is extensive, occurring in 84% of families [8]. The activities and local customs associated with animal production make it impossible to inspect animal carcasses and offal [1],[7],[8],[9], which is a major control option for hydatid disease in those areas and countries where effective control has been achieved [10].

Control of CHD had been grossly neglected in China, and there were no control programs operating until recently. This was due primarily to a weak economy, the resulting lack of funds, and the primitive control approaches used. The situation is now changing because of China's growing economy. The Chinese government has launched an extensive control program that includes dog treatment with praziquantel (PZQ). However, the strategy of choice to reduce the transmission of CHD disease is still problematic [1],[2]. Considering that it is difficult to inspect animal slaughter processes for CHD in hyperendemic areas of China, we designed control measures primarily focusing on eliminating the release of *E. granulosus* eggs into the environment from dogs. Epidemiological surveys have shown that dogs are mainly responsible for the transmission of *E. granulosus* to humans and animals [2],[7],[11],[12],[13]. We simply treated all dogs monthly with PZQ, which did not allow worms to reach maturity even if dogs were infected immediately after a previous treatment, as *E. granulosus* in Xinjiang takes 45 days to become patent [14],[15]. The strategy considerably reduces the amount and expense of control work because dogs are present in smaller numbers than domestic livestock animals, which act as intermediate hosts for transmission of CHD. It is estimated that in western China there are 5 million dogs present compared with to 170 million domestic livestock animals (mainly sheep) [1].

Here we detail a pilot field control project undertaken in 1987–1994 using this approach in two counties (Hutubi and Wensu) in Xinjiang, China. After 4 years of PZQ treatment, the prevalence of *E. granulosus* in dogs was reduced in the two counties from 14.7% and 18.6%, respectively, to zero, and this caused a 90%–100% decrease of CHD in sheep born after commencement of the control program. Monthly dog treatment with PZQ is, therefore, an efficient, highly cost-effective and practicable measure for control of CHD in hyperendemic rural communities in China.

## Materials and Methods

### Ethical approval

All procedures, including monthly treatment of dogs with PZQ, the use of arecoline hydrobromate as a purgative for *E. granulosus* diagnosis, and the humane killing of unwanted and stray dogs were approved by the Ethics Committee of Xinjiang Academy of Animal Science (ECXAAS) and by the Ministry of Agriculture of China and the Committee of Xinjiang Science and Technology, Research and Development. The local governments of Hutubi County and Wensu County agreed to and fully assisted with all the procedures on dogs. The handling, treatment, and culling of all animals was conducted in strict adherence to the Xinjiang Veterinary Institute's guidelines for the experimental use of animals. Questionnaire procedures were approved by ECXAAS; as a large number of subjects were involved, we explained fully the details of the questionnaire and verbal permission was requested from each of the participating interviewees. Participants were free to withdraw from the interview at any time. All interviewees signed the questionnaire when completed.

### Study sites

**Figure 1:**
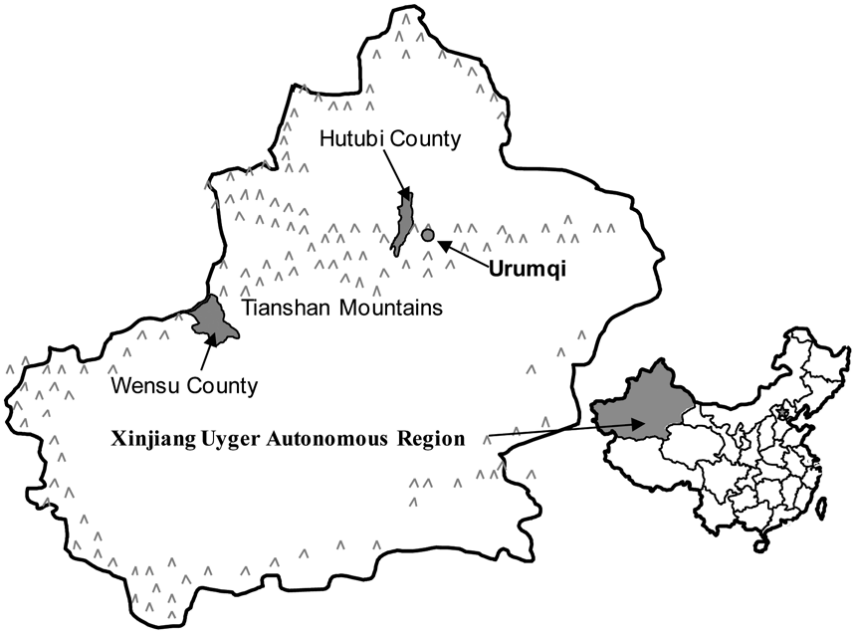
The locations of Hutubi and Wensu Counties, Xinjiang, China. Urumqi is the capital city of Xinjiang.

With support from the Central Chinese Government and the local Government of Xinjiang Uygur Autonomous Region (Xinjiang), the Xinjiang Veterinary Institute (an Institute of the Xinjiang Academy of Animal Science) piloted a program from 1987 to 1994 for controlling CHD in Hutubi and Wensu counties in Xinjiang (Figure 1). The control program design was based on the Xinjiang situation (where there is a large population of Uygur [Muslim Chinese]) in terms of ethnic considerations, the local economic situation, and other factors associated with transmission of CHD there. Hutubi (in North Xinjiang) is predominantly composed of Han Chinese, whereas Wensu (in South Xinjiang) is a Uygur community (Table 1). Previous surveys showed that the two counties were highly endemic for CHD (Figure 1) [7],[16].

**Table 1:** General information about the two counties selected for the pilot control program in Xinjiang.

Category	Subcategory	Hutubi (1987)	Wensu (1990)
Number of households		18,600	33,700
Population	Total	98,504	157,000
	Han (%)	79.3	26.6
	Uygur* (%)	20.7	73.4
Area (km^2^)		9,476	14,309
Number of domestic animals	Total	319,700	495,000
	Sheep (%)	73.8	56.6
	Cattle (%)	13.3	25.3
	Others (%)	12.9	18.1
Number of dogs		15,990	14,490
Dogs/household		0.86	0.43

*: Includes other Muslim ethnic groups.

### Control strategy

The pilot control program was designed for controlling CHD in hyperendemic areas to prevent the release of *E. granulosus* eggs to the environment by monthly treatment of all dogs with PZQ, so that newborn domestic animals were free of infection with CHD. The whole biomass of CHD was decreased by culling old livestock (sheep, cattle, and other husbandry animals) in the endemic areas.

### Control measures

#### 1. Structure of control authorities at the county level and their involvement

**Figure 2:**
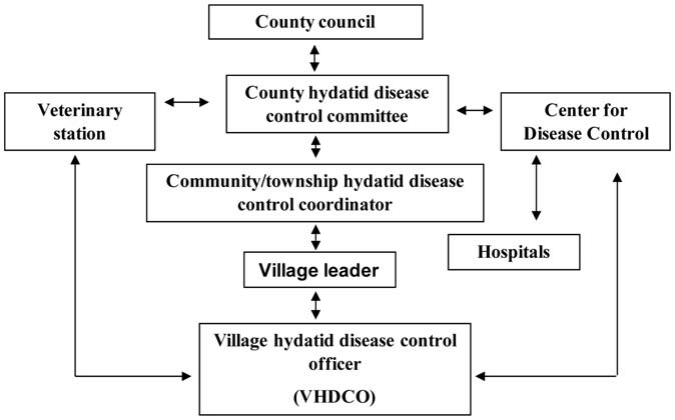
Structural organization for control of hydatid disease at the county level in China.

Figure 2 shows the management structure for the control of CHD established in both counties. The hydatid disease control committee was established at the county level. The duties of the committee were to: (1) undertake leadership of the control program; (2) distribute control program tasks to the relevant departments; (3) monitor control program progress; and (4) reward those personnel who had helped make the control program a success.

One officer was appointed for the control program at the community/township level in both counties. He/she played a liaison role of linking villagers with the county control committee. Every village selected a responsible person as village hydatid disease control officer (VHDCO) who was responsible for implementing control measures in that village. He/she was highly respected by the villagers and was willing to take on the designated duties. Under the supervision of the head of the village, the role of the VHDCO was to: (1) explain the control program to all the villagers; (2) distribute educational materials to villagers; (3) register and dose all dogs in the village monthly with PZQ; (4) check and report stray and unwanted dogs in the village; (5) help police to capture stray/unwanted dogs.

The involvement of veterinary station staff in both counties included: (1) consulting with the county hydatid control committees; (2) providing technical support as required, (3) purchasing and distributing PZQ tablets; (4) monitoring VHDCO tasks; and (5) undertaking baseline and annual surveys of *E. granulosus* in dogs and slaughtered livestock, and annual census of dogs and livestock. Involvement of Centre for Disease Control (CDC) staff in Hutubi and Wensu included: (1) consulting with county hydatid control committees; (2) providing educational materials; (3) responsibility for recording and reporting human CHD cases; (4) use of a questionnaire, with the support of the VHDCOs, as a cheap and effective way to obtain human prevalence (X-ray, ultrasound, and serological testing complemented the questionnaire survey in Hutubi County); (5) monitoring the VHDCOs' tasks; (6) annual reporting of human incidence and changes in status of knowledge, attitudes, and practice with regard to CHD. Local hospitals were involved in human CHD case reporting and treatment.

#### 2. Monthly treatment of all dogs with PZQ

A dog bait tablet containing 100 mg PZQ (Beijing Agrichina Pharmaceutical), which is 100% effective in removing *E. granulosus* worms from the intestines of infected dogs at a dose of 2.1 mg/kg dog body-weight, was used for the control program [17]. The bait was especially designed for treating dogs and was accepted by more than 90% of animals [17]. If a dog rejected the tablet, the VHDCO wrapped the tablet in food and fed the dog with the help of the dog owner. One tablet was used for monthly treatment of all dogs <25 kg in body weight (95% of the dog population in Xinjiang), while two tablets were used for dogs >25 kg [17].

#### 3. Education

The control program team produced a booklet about the disease with cartoon pictures suitable for both students and adults. A booklet was distributed to every family. A colour poster was also designed for every village and was placed on the village notice board. Before the dog treatment day every month, two television programs were broadcast that presented general information on CHD and the control program, respectively.

#### 4. Baseline survey and epidemiological monitoring

Livers and lungs of sheep collected from county villages in Hutubi were monitored at the local county slaughterhouse to determine the prevalence of CHD. Small cysts, especially in sheep aged less than 1 year, that were unsuitable for macroscopic examination were fixed in formalin and sliced into 0.5 mm sections, and any cyst-like material checked microscopically. Any cyst containing both laminated and germinal layers was diagnosed as an *E. granulosus* hydatid cyst. Thirty sheep from each age group were also randomly purchased for examination for further monitoring of CHD prevalence, especially in newly born sheep after commencement of the control program. In Wensu County, as there were no state-owned slaughterhouses and sheep of uncertain origin were killed in the free markets, we purchased sheep directly from the county villages. Three different types of communities were selected based on their village farming practices: mainly agricultural farming but with a small number of livestock, mainly sheep; 50% agricultural farming/50% farming of livestock on pasture; or farming of livestock on pasture only. Ninety sheep were purchased from each of the three community types (Table 2).

**Table 2:** Efficacy of the pilot program for cystic hydatid disease control using dogs and sheep as markers of *Echinococcus granulosus* infection after monthly treatment of dogs with praziquantel in Hutubi County (1987–1990) and Wensu County (1990–1994), Xinjiang, China.

Year^a^	Dog/Sheep – Age (y)	Hutubi County			Wensu County		
		Checked, *n*	Positive, *n* (%)	Reduction %^b^	Checked, *n*	Positive, *n* (%)	Reduction, %^b^
0	Dog	178	33 (18.5)	-	116	17 (14.7)	-
	Sheep - 1	569	473 (83.1)	-	95	77 (81.1)	-
	Sheep - 2	155	136 (87.7)	-	92	64 (69.6)	-
	Sheep - 3	46	42 (91.3)	-	90	62 (68.9)	-
	Sheep - 4	120	113 (94.2)	-	89	67 (75.3)	-
	Sheep ≥5	382	366 (95.8)	-	-	-	-
	**Sub-sheep**	**1,272**	**1,130 (88.8)**	**-**	**366**	**270 (73.8)**	**-**
1^st^	Dog	220	5 (2.3)	87.6	741	12 (1.6)	89.0
	**Sheep - 1**	**30**	**4 (13.3)**	**84.0**	**92**	**17 (18.5)**	**77.2**
2^nd^	Dog	119	0 (0)	100	790	4 (0.5)	96.0
	Sheep - 1	30	4 (13.3)	84.0	102	6 (5.9)	92.7
	Sheep - 2	30	5 (16.7)	81.0	90	7 (7.8)	90.6
	**Sub-sheep**	**60**	**9 (15.0)**	**82.2**	**192**	**13 (6.8)**	**91.0**
3^rd^	Dog	120	0 (0)	100	603	1 (0.2)	98.6
	Sheep - 1	30	1 (3.3)	96.0	93	0 (0)	100
	Sheep - 2	30	1 (3.3)	96.2	90	5 (5.5)	90.6
	Sheep - 3	30	3 (10.0)	89.5	91	6 (6.6)	91.0
	**Sub-sheep**	**90**	**5 (5.6)**	**93.3**	**274**	**11 (4.0)**	**94.5**
4^th^	Dog	-	-	-	111	0 (0)	100
	Sheep -1	-	-	-	101	0 (0)	100
	Sheep - 2	-	-	-	94	1 (1.1)	98.5
	Sheep - 3	-	-	-	83	0 (0)	100
	Sheep - 4	-	-	-	83	4 (4.8)	93.5
	**Sub-sheep**	**-**	**-**	**-**	**361**	**5 (1.4)**	**98.1**

a 0 indicates the baseline survey before dog mass treatment with praziquantel, 1^st^, 2^nd^, 3^rd^ and 4^th^ indicate the first, second, third, and fourth year after control study commencement.

b Reduction % = [prevalence of baseline survey-prevalence of *E. granulosus* in dogs or echinococcosis in new born sheep]/prevalence of baseline survey. All infection rates were significantly less than those obtained during the baseline survey by Z test.

Sub-sheep, sub-total of sheep in bold categories.

Arecoline purgation was used as the method for estimating dog parasite prevalence [18], as it has been used successfully to monitor control programmes in a number of different endemic settings [19]. After dogs were successfully purged, about 30 g of dog feces were collected in a container and the worm burden classified macroscopically as: “−”, negative; “+”, 1–99 worms present; “++”, 100–1,000 worms present; “+++”, more than 1,000 worms present.

The numbers of human CHD cases were obtained by questionnaire analysis of all residents of every village and confirmed by interview with the affected family or the patients themselves.

#### 5. Dog registration and treatment with PZQ

All dogs in each village were registered by the VHDCOs, who informed the villagers that the dogs would be dewormed using the special PZQ bait (described earlier) to remove the parasites causing hydatid disease, and that treatment had to be given every month. The cost of treatment was partly paid for by the dog owners. The VHDCOs were then paid according to the numbers of dogs treated.

#### 6. Training

All leaders and staff in Hutubi and Wensu, including those from each community/township/village involved in the control program, received two hours of special training in workshops that included a 1 hour TV program about CHD and a 1 hour lecture when the details of the control program were explained. After training, a special contract was signed between the county, the community, and the village to clarify the duties. The VHDCOs received 6 hours of training and attended specialized workshops every year of the control program.

#### 7. Elimination of stray and unwanted dogs

A special team, supervised by local police officials, implemented regular elimination of stray and unwanted dogs in each village.

#### 8. Statistical analysis

We compared the infection rates of sheep and dogs randomly selected and surveyed at different time points using the Z-test before and after commencement of the control program.

## Results

### Hyperendemic cystic hydatid disease in Hutubi and Wensu counties

Table 1 shows some general information about the two counties selected for the pilot field control project. Hutubi was confirmed as being predominantly (79.3%) Han Chinese with Wensu County as predominantly (73.4%) Uygur. Sheep were the major livestock animals in the two counties (Table 1). In Hutubi County there the mean number of dogs/household was 0.86, twice that of Wensu County (0.43 dogs/household) (Table 1). Baseline surveys showed that the two counties were hyperendemic for cystic echinococcus (CE). In Hutubi County, the prevalence of echinococcosis in sheep was 88.8% (Table 2). 18.5% of dogs were infected with *E. granulosus*. *Taenia multiceps*, *T. hydatigena*, and *Ancylostoma* spp. were also present in 4.5% of dogs (mean worm burden [MWB] 5.1±5.1), 19.7% (MWB 2.7±1.7), and 32.0% (range 4%–61%), respectively, of 178 examined dogs. In Wensu County, 14.7% of dogs were infected with *E. granulosus* (Table 2). *T. multiceps*, *T. hydatigena*, and *Ancylostoma* spp were also present in 6.0%, 21.5%, and 26.7% of the dogs, respectively. As purging methods cannot show the exact worm burden in dogs, we ranked the number of parasites in 30 grams of feces as an estimate of the total worms present. Most dogs had an *E. granulosus* burden of less than 100 worms. Five dogs in Hutubi County and four in Wensu County harboured heavy infections (≥10,000 worms checked by purging and necropsy); one dog from Hutubi County had a total of 35,430 worms.

A survey of human CE involved a questionnaire survey of two communities (Yuanhuchun and Ershilidian) in Hutubi County. All cases were confirmed by a follow-up family visit. There were 37 CHD cases from 1966 to 1987, and 15 had surgery in 1986–1987 (Table 3), which is 43.8/100,000 annually. Four human CHD cases were found in Hezhuang (Yuanhuchun Community), a village with 652 inhabitants. Two CHD patients were found in Shisihu village (Ershilidian Community), comprising 410 residents in 125 families.

**Table 3:** Investigation of human cystic echinococcosis surgical cases determined by questionnaire in Yuanhuchun Community and Ershilidian Community, Hutubi County, Xinjiang, China.

Duration	Cases
1966–1970	3
1971–1975	3
1976–1980	3
1981–1985	13
1986–1987	15
Total	37

All cases were confirmed by a follow-up visit by the investigating team. In 1987, the two communities had 25,684 inhabitants.

### Efficacy of monthly PZQ dosing of dogs for controlling CHD in newborn sheep

The pilot control program was implemented for 3 years (1987–1990) in Hutubi County and 4 years (1990–1994) in Wensu County to assess whether carefully designed control measures for CHD were practicable, acceptable by the local communities, and successful.

The control measures focused on monthly treatment of dogs with PZQ. Considering that the lowest administrative authority in China is at the village level, we selected one person from each of the study villages as the responsible person to be the village hydatid disease control officer (VHDCO). The VHDCO visited every family in each village every month on the dog dosing day (the 15^th^ of each month) and gave the baited PZQ to dog(s) in the family. After the dog(s) were treated, the dog owner signed a registration book. Annual dog censuses were based on records of dog treatment by VHDCOs in June of each year. There were 15,990 and 14,684 dogs registered for treatment in 1987 and 1988, respectively in Hutubi County. The dog population was reduced to 11,580 in 1989, mainly due to transmission of canine distemper virus between August 1988 and May 1989. In Wensu County, there were 14,490 dogs in 1990 and 13,322 dogs were registered for treatment. There were 14,342 dogs that received treatment in 1991, 14,553 in 1992, and 14,230 in 1993.

In Hutubi the prevalence of dogs infected with *E. granulosus* dropped from 18.5% before commencement of the control measure to zero, paralleling the prevalence in sheep of all ages which was reduced from 88.8% (1,130/1,272) in 1987 to 67.3% (2,259/3,356) in 1988, 58.5% (2,603/4,451) in 1989, and 26.2% (1,135/4,328) in 1990, following yearly surveys from the slaughterhouse in Hutubi County. Infection rates in the newborn sheep of all age groups after the control study had commenced are shown (Table 2). In Wensu, the parasite prevalence in dogs dropped from 14.7% to zero and CE in newborn sheep dropped to 1.4% in the fourth year of the control study (Table 2). As PZQ is a broad-spectrum helminthicide, other highly prevalent dog tapeworms, *T. hydatigina* and *T. multiceps*, also dropped to zero. Of 220 dogs tested, five had *E. granulosus* after 12 months of the control program operating in Hutubi County. Two of dogs harboured mature worms and the remainder had immature *E. granulosus* worms present. In Wensu County, 12 out of 741 dogs were found with *E. granulosus*, and nine dogs had mature worms, indicating that some dogs had not received optimum treatment. After the second year, no mature adult worms were present in *E. granulosus*-positive dogs.

To monitor the progress of the pilot control program, we randomly selected and humanely killed newborn sheep annually from a number of villages to check for the presence of *E. granulosus* in the liver, lung, and other organs. Prevalence of CHD in the newborn sheep was reduced by more than 80% compared with animals of the same age before commencement of the control program in both counties (Table 2).

To limit the numbers of stray dogs (SD) and unwanted dogs (UD), a total of 10,575 dogs were humanely killed in the two counties. In Hutubi County, 656, 489, and 232 dogs were killed, which accounted for 4.1%, 3.3%, and 2.0% of registered dogs in 1987, 1989, and 1990, respectively. In Wensu County, there were more SD and UD in the communities when the control program commenced and 4,021 dogs (including 2,853 SD and 1,168 UD) were killed in 1990. There were 2,205 (245 SD), 1,863 (211 SD), and 1,109 (127 SD) dogs killed in 1991, 1992, and 1993 respectively, which accounted for 15.4%, 12.8%, and 7.8% of registered dogs.

Overall the results showed that the program was operational and successful, and that it had the potential for controlling hydatid disease generally in hyperendemic areas in western China.

### Follow-up of villages in the two counties after completion of the pilot control program

We visited some villages and inspected local slaughterhouses in 2008, 14 and 18 years after completion of the pilot control program in Wensu and Hutubi, respectively. In Hutubi County, we checked 149 sheep of different ages and 4.7% (7/149) had cysts (range 1–10) in the lungs and/or liver. Nine cattle and 77 goats autopsied had no cysts present. Hezhuang village (Hutubi County), which had four human CHD cases before commencement of the control program, had no new HDC cases; the village in the community of Ershilidian with two CHD cases before the control program had one more patient who had surgery in 1999 at age 37. It was not clear whether this patient was infected before or after the control program had commenced.

We visited three villagers in Wensu County who were reported as having CHD, and these were confirmed; they were aged 28, 40, and 55, and had had surgery for CHD in 2000 or 2004. We inspected 2,536 sheep at market slaughter sites; only seven (0.3%) sheep harboured hydatid cysts.

### Cost of control

Considering that cost is a key element in the designing control programs for the field, we estimated the costs for the control measures based on the dog population present based on our study. US$5.2 was spent annually per dog, which allowed smooth operation of the control program in the study areas. This costing covered: (1) PZQ baits (US$1.2/dog); (2) drug delivery by VHDCOs (US$1.5/dog); (3) education costs including booklets and TV broadcasting (equivalent to US$0.5/dog); (4) baseline investigations and progress monitoring (equivalent to US$0.5/dog); (5) administration, including staff employed by county/community authorities (equivalent to US$1.5/dog). Dog owners in the villages in Hutubi and Wensu counties were asked to contribute 50% of the costs for the control program after the extensive education program was introduced. This is not excessive and is affordable, representing only 0.1%–0.2% of the total annual family income. As villagers contributed to the cost of registration and treatment of their dogs they were actively involved in the control program and in monitoring the performance of the VHDCOs; this involvement contributed greatly to the success of the control program.

## Discussion

We have described a pilot program for control of CHD in two counties in Xinjiang, China. Two counties with different ethnic populations were selected to determine whether the same control measures could be used in the different communities. Hutubi County had a majority of Han Chinese, whereas Wensu had a majority of Uygur Muslims. The results showed that control outcomes were similar in both counties, suggesting that the control measures were accepted by the two different ethnic communities.

In the study, we used CHD prevalence in newborn sheep to monitor the efficacy and control progress of control intervention. Dog prevalence of *E. granulosus* as determined by arecoline purgation was also used as a parameter for the evaluation of *E. granulosus* infection prevalence. Although the sensitivity of the purgation is a recognised limitation, its 100% specificity allowed us to compare the efficacy of dog treatment before and after the control program at a time when the more sensitive techniques of copro-ELISA and copro-PCR had not been developed and thus were not available to us for use.

The strategy for control involved monthly treatment of dogs with PZQ and was designed to remove *E. granulosus* before worm maturation so that no eggs would be released into the environment, thereby preventing transmission. As a consequence, there would be no newly born intermediate hosts infected with *E. granulosus*. The disease burden in intermediate hosts was also reduced by culling of old livestock animals. In this respect, the control period depends on the length of the animal culling period. So, the control term depends on the lifespan of domestic animals. Since more than 90% of hydatid cysts in cattle are infertile, these hosts normally play no important role epidemiologically. Sheep play a major role the transmission of CHD since most cysts are fertile. In Xinjiang, the culling age for sheep is 5 years, although a small percentage is raised to 7 years. Consequently, 5 years is a reasonable period for evaluating stage control efficacy, and this period is necessary for a long-term control campaign.

The frequency of dosing dogs was based on studies undertaken on the development of *E. granulosus* in local Xinjiang strains of dogs, which showed animals experimentally infected with protoscoleces of *E. granulosus* first release eggs after 43–45 days [14],[15]. Successful control programs in New Zealand [10],[19] and Rio Negro Province in Argentina [10] treated dogs at an interval of 6 weeks. However, it is problematical to treat dogs for that period in villages in China, as *E. granulosu*s worms will become patent, thereby increasing the risk of infection to intermediate hosts. It can be difficult for control staff to reach village families in semi-nomadic communities during the summer in China when farmers drive their animals to remote pasture areas. Nevertheless, the strategy of monthly dosing dog should be encouraged and is recommended for controlling CHD in these communities, especially during the time when the farmers return to their villages, in order to prevent *E. granulosus* transmission. An extensive education program and a system to remind villagers about monthly treatment can help them to personally treat their own dogs.

We have shown that employing a village hydatid disease control officer (VHDCO) in each village was an optimal way to deliver PZQ prior to the proposed control program [7],[20],[21]. We thus used monthly treatment and extended this to the two counties. Another advantage of the VHDCO system is proper registration of dogs, which is a very important step for controlling CHD. We found 78% of dogs were less than 3 years old and 45% of dogs were less than 1 year old in study villages, indicating that dog culling was frequent. As the VHDCO knew every family in the village, it was easy for him/her to register dogs.

Use of baited tablets is important for high-quality treatment of dogs. We used normal PZQ tablets to treat dogs before entering the villages for the control program study. We found almost no dogs took the tablets, which persuaded us to use baited PZQ tablets for dog treatment. The baited tablet was taken by 90% of dogs over a period of 12 months of treatment in Hutubi County (unpublished data). Notably, we recently tested the formulation in a number of villages in Nagchu County in Tibet, where 97% of dogs took the baited tablet; only a few sick dogs did not take the bait. In our control study, we determined that about 10% of dogs rejected the tablet due sickness or for some other reason. These dogs were treated successfully by VHDCOs wrapping the tablets in food, which was then fed to these animals.

Although challenging work, culling stray and unwanted dogs is very important for CHD control. We sacrificed almost all unwanted and stray dogs in the two counties in the pilot study. We found a large difference between the two counties in terms of the number of stray and unwanted dogs. There were more stray dogs around the communities in Wensu County prior to commencement of the control program. This was due to the majority of the population being Uygur Muslims, to whom dogs are dirty animals and dog meat is forbidden for consumption. In Han Chinese Hutubi County, in contrast, dogs were regarded as domestic animals that can be raised for meat consumption. We believe this is the main reason Hutubi County had fewer stray and unwanted dogs than Wensu County. After the control program commenced, the number of stray and unwanted dogs was maintained at 8%–15% in Wensu County.

This pilot control program focused in Xinjiang showed that the strategy to prevent eggs being released from dogs to the external environment by treating animals before adult tapeworms are patent can cut the transmission of *E. granulosus* and reduce considerably the level of hyperendemic CHD. Dog treatment with PZQ given monthly is an efficient and highly cost-effective (we estimate direct annual cost of PZQ in 2009 is US$1.2 per dog) and practicable measure for rural communities. As a result, the Chinese Ministry of Health has launched an extensive CHD control program, initiated in 2005 in ten counties in Ganzi Tibetan Prefecture, Sichuan province [22], and extended to 117 counties in western China in 2009 (Dr. Weiping Wu, personal communication).

## Supporting Information

Checklist S1 STROBE checklist.
(0.08 MB DOC)

## References

[1] Chi PS, Fan YL, Zhang WB, Zhang ZZ, Alili H (1989). The epidemic situations of cystic echinococcosis in China.. Xinjiang Agricultural Sciences.

[2] Li TY, Qiu JM, Yang W, Craig PS, Chen XW (2005). Echinococcosis in Tibetan populations, western Sichuan Province, China.. Emerg Infect Dis.

[3] He T-L (2000). The prevalence and prevention of hydatid disease in Qinghai Province.. Chinese Journal of Zoonoses.

[4] Wang H (1994). The epidemiological situation of *Echinococcus*/echinococcosis in Qinghai Plateau.. Chinese Qinghai Journal of Animal and Veterinary Sciences.

[5] Li L, Xia Q, Fu DR, Xiao D, Duan MX (2005). An epidemiological survey on echinococcosis in populations in Ningxia Hui Autonomous Region.. Chinese Journal of Zoonoses.

[6] Wang JG, Zhang CJ (2000). The Epidemiological investigation on hydatidosis in Gansu province.. Endemic Diseases Bulletin.

[7] Chi P, Zhang W, Zhang Z, Hasyet M, Liu F (1990). Cystic echinococcosis in the Xinjiang/Uygur Autonomous Region, People's Republic of China. I. Demographic and epidemiologic data.. Trop Med Parasitol.

[8] Zhang WB, Zhanh ZZ, Alili H, Chi PS (1991). An investigation on the social factors influencing the transmission of hydatidosis in rural area in Xinjiang, China.. Endemic Diseases Bulletin.

[9] Zhang WB, Zhang ZZ, Alili H, Chi PS (1994). An investigation of factors influencing the hyperendemic of echinococcosis in Xinjiang.. Chinese Journal of Zoonoses.

[10] Craig PS, Larrieu E (2006). Control of cystic echinococcosis/hydatidosis: 1863–2002.. Adv Parasitol.

[11] Wang YH, Rogan MT, Vuitton DA, Wen H, Bartholomot B (2001). Cystic echinococcosis in semi-nomadic pastoral communities in north- west China.. Trans R Soc Trop Med Hyg.

[12] Ma SM, Wang H, Li WM (2006). Analysis on endemic status on echinococcosis in Qinghai Province.. Journal of Tropical Medicine.

[13] He J-G, Qiu J-M, Liu F-J, Chen X-W, Liu D-L (2000). The epidemiological study on hydatid disease in west Sichuan. II. Prevalence of cystic and alveolar echinococcosis in animals.. Chinese Journal of Zoonoses.

[14] Zhang WB, Alili H, Zhang ZZ, Chi PS (1991). Development and sexual maturation of *Echinococcus granulosus* in local strain of dogs in the Xinjiang, China.. Chinese Journal of Veterinary Science & Technology.

[15] Zhang YL, Ha J, Alili H, Chi PS, Ba K (1989). Development of *Echinococcus granulosus* in dogs.. Xinjiang Agricultural Sciences.

[16] Wu P (1994). Application of hydatid control measures used in Hutubi County.. Cao Shi Jia Xiu.

[17] Zhang WB, Zhang ZZ, Alili H, Wang WM, Chi PS (1990). The efficiency of Praziquantel baits against *Echinococcus granulosus* in dogs.. Endemic Diseases Bulletin.

[18] Andersen FL (1987). Procedure for purging dogs for detection of *Echinococcus granulosus*.. Endemic Disease Bulletin.

[19] Gemmell MA, Roberts MG, Beard TC, Campano Diaz S, Lawson JR, Eckert J, Gemmell MA, Meslin F-X, Pawlowski ZS (2001). Control of *Echinococcus granulosus*..

[20] Andersen FL, Tolley HD, Schantz PM, Chi P, Liu F (1991). Cystic echinococcosis in the Xinjiang/Uygur Autonomous Region, People's Republic of China. II. Comparison of three levels of a local preventive and control program.. Trop Med Parasitol.

[21] Andersen FL, Andersen FL, Chai J-J, Liu F-J (1993). General introduction to cystic echinococcosis and description of cooperative research efforts in the Xinjiang Uyger Autonomous Region, PRC..

[22] Yu SH (2008). Global progress of echinococcosis control and an insight to the national control program.. Chinese Journal of Parasitology and Parasite Diseases.

